# Reduced Fluoroquinolone Susceptibility in *Salmonella enterica* Isolates from Travelers, Finland

**DOI:** 10.3201/eid1505.080849

**Published:** 2009-05

**Authors:** Marianne M. Lindgren, Pirkko Kotilainen, Pentti Huovinen, Saija Hurme, Susanna Lukinmaa, Mark A. Webber, Laura J.V. Piddock, Anja Siitonen, Antti J. Hakanen

**Affiliations:** National Institute of Health and Welfare, Turku, Finland (M.M. Lindgren, P. Kotilainen, P. Huovinen, A.J. Hakanen); Turku University Hospital, Turku (P. Kotilainen, A.J. Hakanen); University of Turku, Turku (S. Hurme); National Institute of Health and Welfare, Helsinki, Finland (S. Lukinmaa, A. Siitonen); University of Birmingham, Birmingham, UK (M.A. Webber, L.J.V. Piddock)

**Keywords:** Antimicrobial resistance, enteric infections, nonclonal, reduced susceptibility, Salmonella enterica, serovar, travelers’ diarrhea, Finland, dispatch

## Abstract

We tested the fluoroquinolone susceptibility of 499 *Salmonella enterica* isolates collected from travelers returning to Finland during 2003–2007. Among isolates from travelers to Thailand and Malaysia, reduced fluoroquinolone susceptibility decreased from 65% to 22% (p = 0.002). All isolates showing nonclassical quinolone resistance were from travelers to these 2 countries.

Fluoroquinolones are the most commonly used antimicrobial agents for the treatment of salmonellosis in adult patients (*1*). The proportion of nontyphoidal strains of *Salmonella enterica* with reduced fluoroquinolone susceptibility has increased during recent years in many countries (*1*–*3*). In Finland, fluoroquinolone susceptibility of salmonella has been surveyed since 1995 by analyzing isolates from patients who acquired the disease either at home or abroad. From 1995 through 2004, reduced fluoroquinolone susceptibility (MIC >0.125 µg/mL) increased significantly (4.0% to 39%) among all foreign *Salmonella* isolates (*3*,*4*). The increase was most prominent among isolates from Southeast Asia, especially Thailand (*4*).

Until 2002, all *Salmonella* isolates worldwide with reduced ciprofloxacin susceptibility were uniformly resistant to nalidixic acid (*3*–*5*); i.e., they exhibited the conventional quinolone resistance phenotype. In 2003, we identified *Salmonella* isolates that showed reduced susceptibility to ciprofloxacin but were either susceptible (MIC <32 µg/mL) or only low-level resistant (MIC = 32 µg/mL) to nalidixic acid. All *Salmonella* isolates with this nonclassical quinolone resistance phenotype were from travelers returning from Thailand or Malaysia (*6*). We undertook this study to survey the recent incidence of reduced fluoroquinolone susceptibility among nontyphoidal strains of *S. enterica* acquired by Finnish travelers abroad and to define the epidemiology of the nonclassical quinolone-resistant *Salmonella* population.

## The Study

Our study included 499 *S. enterica* isolates collected during 2003–2007 from Finnish travelers returning from abroad; due to a technical error, in 2004 only 99 foreign *Salmonella* isolates were sent to us from the National Salmonella Reference Center. The first 100 foreign *Salmonella* isolates identified during January of each year were collected. An isolate was designated to be of foreign origin if the patient had reported travel abroad during the month before the specimen was obtained. Epidemiologic information regarding travel destination was collected from the forms accompanying each isolate.

MICs of ciprofloxacin and nalidixic acid for the isolates were determined by using plate agar dilution (*7*). The MIC breakpoint value for reduced ciprofloxacin susceptibility was chosen as >0.125 µg/mL on the basis of earlier publications (*4*,*5*,*8*). The breakpoints for nalidixic acid were 16 µg/mL for susceptibility and 32 µg/mL for resistance, according to Clinical and Laboratory Standards Institute guidelines (*7*).

Pulsed-field gel electrophoresis (PFGE) was performed using PulseNet standardized protocol with few modifications (*9*). We considered any difference between 2 profiles to be sufficient to distinguish 2 different PFGE profiles.

Data concerning the number of travelers from Finland to countries of interest (i.e., countries with *Salmonella* isolates showing reduced fluoroquinolone susceptibility) during the study months were received from Statistics Finland (www.stat.fi). Susceptibility data were analyzed by using WHONET5.4. For statistical analyses, we summarized data on the basis of the number and proportion of *Salmonella* isolates with reduced fluoroquinolone susceptibiity. The trend over years was analyzed using a logistic regression model with year as a covariate; p<0.05 was considered significant. Statistical analyses were performed using SAS for Windows version 9.1.3 (SAS Institute Inc., Cary, NC, USA).

**Figure 1:**
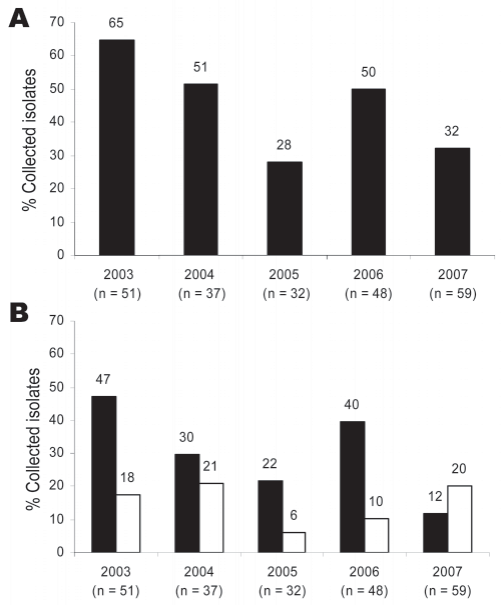
Ciprofloxacin susceptibility and quinolone resistance in 227 *Salmonella* *enterica* isolates collected from travelers returning to Finland from Thailand or Malaysia, 2003–2007. A) Percentage of isolates showing reduced ciprofloxacin susceptibility (black bars, MIC >0.125 µg/mL, p = 0.002). B) Percentage of isolates showing conventional (black bars, MIC of nalidixic acid >32 µg/mL, p = 0.0014) or nonclassical (white bars, MIC of nalidixic acid <32 µg/mL, p = 0.878) quinolone resistance phenotype.

Of the 499 *S. enterica* isolates collected, 227 came from travelers returning to Finland from Thailand or Malaysia (Table). Among all *Salmonella* isolates, reduced fluoroquinolone susceptibility decreased from 48% in 2003 to 34% in 2007 (p = 0.029). Among the isolates from Thailand and Malaysia, the decrease was 65% to 32% (p = 0.002) (Figure 1, panel A). However, when excluding isolates from Thailand and Malaysia, the reduced fluoroquinolone susceptibility remained fairly stable from 2003 to 2007 (31% vs. 37%; p = 0.787).

**Table:** Quinolone susceptibility of 499 *Salmonella* isolates collected from travelers returning to Finland, by country visited, 2003−2007*

Country visited	No. (%) isolates	No. isolates with CIP MIC >0.125 µg/mL	No. isolates with NAL MIC >32 µg/mL	No. isolates with NAL MIC <32 µg/mL	No. trips from Finland during study months†
Thailand‡	212 (42.5)	98	67	31	122,472
Spain§	66 (13.2)	25	25	0	426,822
Brazil	41 (8.2)	0	0	0	25,009
Egypt	40 (8.0)	17	17	0	47,291
India	32 (6.4)	7	7	0	26,233
Malaysia	15 (3.0)	6	1	5	2,597
Vietnam	9 (1.8)	2	2	0	7,555
Tanzania	9 (1.8)	2	2	0	1,259
Portugal	8 (1.6)	7	7	0	35,899
Morocco	5 (1.0)	3	3	0	8,708
Other areas	62 (12.4)	14	14	0	
Total	499 (100)	181	145	36	

*CIP, ciprofloxacin; NAL, nalidixic acid. †Data collected from statistics reports, Finland, based on the no. of Finnish travelers to these countries. ‡No. Finnish travelers to Thailand from 2003 to 2007: 21,873; 22,882; 17,661; 21,941; and 38,115, respectively. §Includes Canary Islands.

Among all *Salmonella* isolates, conventional quinolone resistance decreased significantly during the study, from 39% in 2003 to 22% in 2007 (p = 0.012). This decrease was even more conspicuous among isolates from Thailand and Malaysia (47% vs. 12%; p = 0.0014) (Figure 1, panel B).

The nonclassical quinolone resistance phenotype first appeared in 2003. Subsequently, 36 *Salmonella* isolates showing this resistance pattern have been identified. From 2003 through 2007, the yearly proportions of *Salmonella* isolates showing the nonclassical quinolone resistance phenotype were 9%, 8%, 2%, 5%, and 12%, respectively; there was no significant difference from year to year (p = 0.720). Among the isolates from Thailand and Malaysia, the nonclassical quinolone resistance varied; however, the difference from year to year was not significant (p = 0.878) (Figure 1, panel B).

The 499 *Salmonella* isolates in this study were collected from travelers returning from 43 different countries; isolates with reduced ciprofloxacin susceptibility were collected from travelers returning from 18 countries (Table). All 36 isolates showing the nonclassical resistance phenotype were from Thailand or Malaysia; 47% of the isolates showing the conventional quinolone resistance phenotype were from these 2 countries.

**Figure 2:**
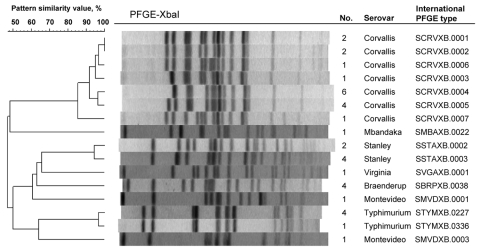
Dendrogram showing the clonal relationships among 36 isolates of *Salmonella* *enterica* collected from travelers returning to Finland from Thailand or Malaysia, 2003–2007. These isolates showed the nonclassical quinolone resistance phenotype (i.e., reduced susceptibility to ciprofloxacin [MIC >0.125 µg/mL] and susceptibility or low-level resistance to nalidixic acid [MIC <32 µg/mL]). No., number of *Salmonella* isolates belonging to a certain pulsed-field gel electrophoresis (PFGE) pattern.

The nonclassical quinolone resistance phenotype was found in 7 different serovars, of which *S*. *enterica* serovar Corvallis and *S*. *enterica* serovar Stanley were the most prevalent; among the conventional quinolone resistance phenotype, *S*. *enteritidis* and *S*. *virchow* were the most prevalent. The 36 isolates belonging to the nonclassical phenotype were distinguished by PFGE to 16 different PFGE patterns (Figure 2).

## Conclusions

Reduced fluoroquinolone susceptibility among nontyphoidal strains of *S. enterica* among travelers from Finland, as a whole, decreased significantly during the study period, 2003–2007 after having constantly increased for several years (*3*,*4*). The significant decrease (p = 0.029) from 2003 to 2007 was driven by isolates from Southeast Asia; the proportion of resistant strains from the other travel destinations remained fairly stable. Notably, this recent decreasing trend in resistance was not linked with reduced travel to Thailand, as the number of tourists from Finland going to Thailand more than doubled from 2005 to 2007 (Table).

In addition, after the emergence of the nonclassical quinolone resistance phenotype among *S. enterica* in Thailand and Malaysia, these isolates have persisted in that area. Our study shows a minor variation in the proportion of this resistance phenotype among the whole quinolone-resistant population. For example, in 2005 the proportion of the nonclassical phenotype isolates was only 6%, but in that year, the proportion of all isolates with reduced ciprofloxacin susceptibility was small. This result is most likely due to the collection of the foreign isolates soon after the tsunami catastrophe in December 2004; travelers from Finland took >20% fewer trips to Thailand in 2005 than in 2004 (Table).

All of the *Salmonella* isolates showing the nonclassical quinolone resistance phenotype were from Thailand or Malaysia. Despite this geographic stability, isolates of the nonclassical phenotype were nonclonal, as shown by PFGE. These findings provoke the question of whether the emergence, persistence, and confinement of those isolates in this area might have something to do with the living conditions of the residing population. In 2007, the proportion of the nonclassical phenotype surpassed that of the conventional phenotype for the first time (20% vs. 12%; Figure 1, panel B). This increase in nonclassical phenotypes may be an emerging trend that needs to be under close surveillance.

The nonclassical quinolone-resistant population may prove hard to identify in those microbiological laboratories that use only nalidixic acid to screen for reduced fluoroquinolone susceptibility in salmonella isolates. It is to be expected that this screening approach may fail due to susceptibility or only low-level resistance to nalidixic acid in these isolates (*10*–*12*). Isolates collected from travelers returning from Thailand or Malaysia should especially be examined for fluoroquinolone susceptibility because nalidixic acid screening test results may no longer be predictive of fluoroquinolone resistance. At the present time, the nonclassical phenotype appears to be mainly confined to Thailand and Malaysia (*10*,*11*), but given the continuous increase in global travel, these isolates may emerge in other parts of the world.

## References

[1] Stevenson JE, Gay K, Barrett TJ, Medalla F, Chiller TM, Angulo FJ. Increase in nalidixic acid resistance among non-typhi *Salmonella enterica* isolates in the United States from 1996 to 2003. Antimicrob Agents Chemother. 2007;51:195–7. 10.1128/AAC.00222-06 17088493 PMC1797669

[2] Chau TT, Campbell JI, Galindo CM, Van Minh Hoang N, Diep TS, Nga TT, Antimicrobial drug resistance of *Salmonella enterica* serovar Typhi in Asia and molecular mechanism of reduced susceptibility to the fluoroquinolones. Antimicrob Agents Chemother. 2007;51:4315–23. 10.1128/AAC.00294-07 17908946 PMC2167998

[3] Hakanen AJ, Kotilainen P, Pitkänen S, Huikko S, Siitonen A, Huovinen P. Reduction in fluoroquinolone susceptibility among non-typhoidal strains of *Salmonella enterica* isolated from Finnish patients. J Antimicrob Chemother. 2006;57:569–72. 10.1093/jac/dkl002 16436543

[4] Hakanen A, Kotilainen P, Huovinen P, Helenius H, Siitonen A. Reduced fluoroquinolone susceptibility in *Salmonella enterica* serotypes in travelers returning from Southeast Asia. Emerg Infect Dis. 2001;7:996–1003. 11747728 10.3201/eid0706.010613 PMC2631904

[5] Hakanen A, Kotilainen P, Jalava J, Siitonen A, Huovinen P. Detection of decreased fluoroquinolone susceptibility in salmonellas and validation of nalidixic acid screening test. J Clin Microbiol. 1999;37:3572–7. 10523554 10.1128/jcm.37.11.3572-3577.1999 PMC85694

[6] Hakanen AJ, Lindgren M, Huovinen P, Jalava J, Siitonen A, Kotilainen P. New quinolone resistance phenomenon in *Salmonella enterica*: nalidixic acid-susceptible isolates with reduced fluoroquinolone susceptibility. J Clin Microbiol. 2005;43:5775–8. 10.1128/JCM.43.11.5775-5778.2005 16272517 PMC1287832

[7] Mølbak K, Baggesen DL, Aarestrup FM, Ebbesen JM, Engberg J, Frydendahl K, An outbreak of multidrug-resistant, quinolone-resistant *Salmonella enterica* serotype Typhimurium DT104. N Engl J Med. 1999;341:1420–5. 10.1056/NEJM199911043411902 10547404

[8] Clinical and Laboratory Standards Institute. Performance Standards for Antimicrobial Susceptibility Testing: Seventeenth Informational Supplement. Vol. 27. Wayne (PA): The Committee; 2007. Document no. M-100-S17.

[9] Lukinmaa S, Nakari UM, Liimatainen A, Siitonen A. Genomic diversity within phage types of *Salmonella enterica* ssp. enterica serotypes Enteritidis and Typhimurium. Foodborne Pathog Dis. 2006;3:97–105. 10.1089/fpd.2006.3.97 16602985

[10] Cavaco LM, Hendriksen RS, Aarestrup FM. Plasmid-mediated quinolone resistance determinant *qnrS1* detected in *Salmonella enterica* serovar Corvallis strains isolated in Denmark and Thailand. J Antimicrob Chemother. 2007;60:704–6. 10.1093/jac/dkm261 17635876

[11] Hopkins KL, Day M, Threlfall EJ. Plasmid-mediated quinolone resistance in *Salmonella enterica*, United Kindom. Emerg Infect Dis. 2008;14:340–2. 10.3201/eid1402.070573 18258138 PMC2600194

[12] Threlfall EJ, Ward LR, Skinner JA, Smiths HR, Lacey S. Ciprofloxacin-resistant *Salmonella typhi* and treatment failure. Lancet. 1999;353:1590–1. 10.1016/S0140-6736(99)01001-6 10334265

